# Magnetic resonance imaging of glioma with novel APTS-coated superparamagnetic iron oxide nanoparticles

**DOI:** 10.1186/1556-276X-9-304

**Published:** 2014-06-15

**Authors:** Kangan Li, Mingwu Shen, Linfeng Zheng, Jinglong Zhao, Qimeng Quan, Xiangyang Shi, Guixiang Zhang

**Affiliations:** 1 Department of Radiology, Shanghai First People’s Hospital, Shanghai Jiaotong University School of Medicine, Shanghai 200080, People’s Republic of China; 2 College of Chemistry, Chemical Engineering and Biotechnology, Donghua University, Shanghai 201620, People’s Republic of China

**Keywords:** Iron oxide nanoparticles, 3-Aminopropyltrimethoxysilane, Magnetic resonance imaging, Tumor cells, Glioma

## Abstract

We report *in vitro* and *in vivo* magnetic resonance (MR) imaging of C6 glioma cells with a novel acetylated 3-aminopropyltrimethoxysilane (APTS)-coated iron oxide nanoparticles (Fe_3_O_4_ NPs). In the present study, APTS-coated Fe_3_O_4_ NPs were formed via a one-step hydrothermal approach and then chemically modified with acetic anhydride to generate surface charge-neutralized NPs. Prussian blue staining and transmission electron microscopy (TEM) data showed that acetylated APTS-coated Fe_3_O_4_ NPs can be taken up by cells. Combined morphological observation, cell viability, and flow cytometric analysis of the cell cycle indicated that the acetylated APTS-coated Fe_3_O_4_ NPs did not significantly affect cell morphology, viability, or cell cycle, indicating their good biocompatibility. Finally, the acetylated APTS-coated Fe_3_O_4_ nanoparticles were used in magnetic resonance imaging of C6 glioma. Our results showed that the developed acetylated APTS-coated Fe_3_O_4_ NPs can be used as an effective labeling agent to detect C6 glioma cells *in vitro* and *in vivo* for MR imaging. The results from the present study indicate that the developed acetylated APTS-coated Fe_3_O_4_ NPs have a potential application in MR imaging.

## Background

The molecular imaging (MI) of tumors has recently gained widespread use [1-4] due to its ability to facilitate quantitative and repetitive imaging of targeted molecules and biological processes in living organisms [2,5,6]. Contrast agents are generally required for high-quality MI diagnosis. Advances in nanotechnology enable the development of various nanoparticles (NPs) as contrast agents for effective MI in the diagnosis or analysis of diseases. Superparamagnetic iron oxide nanoparticles (SPIONs) are a promising form of imaging probe that can accumulated in cells and generate a strong magnetic resonance (MR) imaging contrast in T2- or T2*-weighted images [7]. To date, SPIONs have been used to investigate several pathophysiological processes in tumor cells [8,9], transplanted cells [1,7,10], or precursor cells *in vivo*[11-13].

SPION probes are generally comprised of superparamagnetic iron oxide cores of magnetite or maghemite NPs encased in various coatings. Cellular uptake of SPIONs may be achieved by phagocytosis, macropinocytosis, or receptor-mediated endocytosis [2,14,15]. For effective MR imaging applications, synthesizing SPIONs with controllable sizes and surface functionalities is important, given that the *in vivo* biomedical performance, especially the opsonization, pharmacokinetics, and biodistribution, of the SPIONs can be directly impacted by the particle's size and surface modifications [16]. Various methods have been employed to synthesize SPIONs with controllable size, such as controlled co-precipitation of Fe(II) and Fe(III) ions at an elevated temperature [17], successive reduction-oxidation process in a reverse micelle system [18], thermal decomposition [19], and a hydrothermal method under higher pressures [20]. To make SPIONs with good water dispersity and desired surface functionality for biomedical applications, surfactant molecules [21,22], silane agents [23-25] or other small molecular ligands [9,26-28], polyethylene glycol (PEG) derivatives [29,30], and dendrimers [15,31,32] have been used to modify SPIONs using either *in situ* modifications or post-modification approaches.

In our previous work, we adopted a simple one-step 3-aminopropyltrimethoxysilane (APTS)-assisted hydrothermal approach to synthesize APTS-coated Fe_3_O_4_ NPs with reactive surface amine groups [33]. The APTS modification endowed Fe_3_O_4_ NPs with an excellent water dispersibility and colloidal stability. Additionally, these APTS-coated Fe_3_O_4_ NPs can be further functionalized with acetyl groups with neutral surface potential following the reaction of the surface APTS amines with acetic anhydride. Our results suggest that the presence of APTS molecules not only enables efficient APTS coating of the particles with reactive amine groups but also significantly limits the particle growth. This prior success led us to hypothesize that acetylated APTS-coated Fe_3_O_4_ NPs may serve as a labeling agent for MR imaging of cancer cells both *in vitro* and *in vivo*.

In the present study, we synthesized acetylated APTS-coated Fe_3_O_4_ NPs with a mean diameter of 6.5 nm, similar to our previous report [33]. The formed acetylated APTS-coated Fe_3_O_4_ NPs were used as a labeling agent for *in vitro* and *in vivo* MR imaging of C6 glioma cells. The cellular uptake of the acetylated APTS-coated Fe_3_O_4_ NPs was confirmed by Prussian blue staining and transmission electron microscopy (TEM) imaging. Combined morphological observation of the cells, a 3-(4,5-dimethylthiazol-2-yl)-2,5-diphenyltetrazolium bromide (MTT) assay of cell viability, and flow cytometric analysis of the cell cycle were used to evaluate the cytotoxicity of the acetylated APTS-coated Fe_3_O_4_ NPs.

## Methods

### Materials

Ferrous chloride tetrahydrate (FeCl_2_ · 4H_2_O >99%), ammonia (28% to 30% NH_3_ in aqueous solution), triethylamine, acetic anhydride, and dimethyl sulfoxide (DMSO) were purchased from Sinopharm Chemical Reagent Co., Ltd (Shanghai, China). The APTS and acetic anhydride were from Acros Organics (Geel, Belgium). C6 glioma cells (a rat C6 glioma cell line) were purchased from the Institute of Biochemistry and Cell Biology at the Chinese Academy of Sciences (Shanghai, China). RPMI 1640 medium, fetal bovine serum (FBS), penicillin, and streptomycin were purchased from Hangzhou Jinuo Biomedical Technology (Hangzhou, China). The MTT was acquired from Shanghai Sangon Biological Engineering Technology and Services Co., Ltd (Shanghai, China). The water that was used in all of the experiments was purified using a Milli-Q Plus 185 water purification system (Millipore, Bedford, MA, USA) with a resistivity that was higher than 18.2 MΩ cm.

### The synthesis of acetylated APTS-coated Fe_3_O_4_ NPs

APTS-coated Fe_3_O_4_ NPs were synthesized using a hydrothermal approach, which was described in our previous study [20,33]. Typically, FeCl_2_ · 4H_2_O (1.25 g) was dissolved in 7.75 mL water. Under vigorous stirring, ammonium hydroxide (6.25 mL) was added, and the suspension was continuously stirred in air for 10 min. Next, 2.5 mL APTS was added, and the reaction mixture was autoclaved (KH-50 Autoclave, Shanghai Yuying Instrument Co., Ltd., Shanghai, China) in a sealed pressure vessel with a volume of 50 mL at 134°C. After 3 h, the reaction mixture was cooled to room temperature. The black precipitate was collected and purified with water five times and with ethanol twice via a centrifugation-dispersion process (5,000 rpm, 10 min) to remove excess reactants. Lastly, the obtained APTS-coated Fe_3_O_4_ NPs were dispersed in ethanol.

The amine groups on the surface of the APTS-coated Fe_3_O_4_ NPs were further acetylated via a reaction with acetic anhydride, following the protocols described in our previous study [33]. Briefly, 1 mL of triethylamine was added to the APTS-coated Fe_3_O_4_ NPs (6 mg) solution that was dispersed in ethanol (5 mL), and the solution was thoroughly mixed. A DMSO solution (5 mL) that contained acetic anhydride (1 mL) was added dropwise into the solution of APTS-coated Fe_3_O_4_ NPs, which was mixed with triethylamine while being stirred vigorously. The mixture was allowed to react for 24 h. The DMSO, excess reactants, and by-products were removed from the mixture by a centrifugation/washing/dispersion step that was repeated five times to obtain acetylated APTS-coated Fe_3_O_4_ NPs dispersed in water.

### Characterization techniques

The morphology of the formed acetylated APTS-coated Fe_3_O_4_ NPs was observed by TEM imaging using a JEOL 2010 F analytical electron microscope (Akishima-shi, Japan) that operated at 200 kV. The TEM sample was prepared by placing one drop of diluted suspension of acetylated APTS-coated Fe_3_O_4_ NPs (5 μL) onto a 200-mesh carbon-coated copper grid and air-dried prior to measurement. The size of the NPs was measured using ImageJ 1.40G image analysis software (http://rsb.info.nih.gov/ij/download.html). A minimum of 200 randomly selected NPs in different TEM images were analyzed for each sample to acquire the size distribution histogram.

The transverse relaxometry was performed using a Signa HDxt 3.0 T superconductor magnetic resonance system (GE Medical Systems, Milwaukee, WI, USA) with a wrist receiver coil. For the MR transverse relaxometry measurements, the acetylated APTS-coated Fe_3_O_4_ NPs (1 mL) that were dispersed in water at different concentrations were separately added to 1.5-mL Eppendorf tubes. The transverse relaxation times (*T*_2_) were measured using a multi-echo fast spin echo (MFSE) sequence. A total of eight echoes were used with the following parameters: repetition time (TR) = 500 ms, echo time (TE) = 21.9 ms, flip angle = 90°, resolution = 256 × 256, section thickness = 2 mm, and field of view (FOV) = 80 × 80 mm. The *R*_2_ mapping was performed using a workstation running Functool 4.5.3 (GE Medical Systems, Milwaukee, WI, USA). The transverse relaxivities (*R*_2_, 1/*T*_2_) were determined using a linear fit of 1/*T*_2_ as a function of the Fe concentration of the particles. The Fe concentration of the acetylated APTS-coated Fe_3_O_4_ NPs was analyzed using Prodigy inductively coupled plasma-atomic emission spectroscopy (ICP-AES) (Teledyne Leeman Labs, Hudson, NH, USA) following aqua regia treatment.

### Cytotoxicity of acetylated APTS-coated Fe_3_O_4_ NPs

The C6 glioma cells were continuously grown in a 50-mL culture flask in regular RPMI 1640 medium that was supplemented with 10% heat-inactivated FBS, 100 U/mL penicillin, and 100 U/mL streptomycin. A 3-(4,5-dimethylthiazol-2-yl)-2,5-diphenyltetrazolium bromide (MTT) assay was used to quantify the viability of the cells upon treatment with the acetylated APTS-coated Fe_3_O_4_ NPs. Briefly, 1 × 10^4^ C6 glioma cells per well were seeded into a 96-well plate. Following overnight incubation to bring the cells to confluence, the medium was replaced with fresh medium that contained the acetylated APTS-coated Fe_3_O_4_ NPs at different concentrations (0, 1, 10, 25, 50, and 100 μg/mL). After 24 h of incubation at 37°C, the metabolically active cells were subsequently detected by adding MTT to each well. The assays were performed according to the manufacturer's instructions, and the absorbance of each well was measured using a Thermo Scientific Multiskan MK3 ELISA reader (Thermo Scientific, Waltham, MA, USA) at 570 nm. The mean and the standard error mean (SEM) for the triplicate wells were reported and normalized. One-way analysis of variance (ANOVA) statistical analyses were performed to detect the difference between the cells that were incubated with different concentrations of acetylated APTS-coated Fe_3_O_4_ NPs and the control cells, which were treated with phosphate-buffered saline (PBS) buffer. The statistical significance level was set to 0.05.

The cytotoxicity of the acetylated APTS-coated Fe_3_O_4_ NPs was further examined using flow cytometric analysis of the cell cycle and apoptosis [34]. C6 glioma cells were seeded in six-well cell culture plates at a density of 3 × 10^5^ cells per well in quadruplet and were allowed to grow to confluence for 24 h. Next, after replacing the medium with fresh medium that contained different concentrations of acetylated APTS-coated Fe_3_O_4_ NPs (0, 50, and 100 μg/mL), the cells were incubated for 4 h at 37°C in a CO_2_ incubator. Following the treatment, the cells were harvested by trypsinization and centrifugation, washed with PBS buffer, and fixed in citrate buffer for 2 h. The cells were later centrifuged to remove the citrate buffer and resuspended with PBS buffer with a cell concentration of 1 × 10^6^ cells/mL. The cell suspensions were incubated with trypsinogen for 3 min and then incubated with RNase for 3 min. Subsequently, the cells were stained with propidium iodide (PI) for 15 min, and the PI-stained cells were then counted using flow cytometry (FACSCalibur, Becton Dickinson, Franklin Lakes, NJ, USA) in the red (FL2) channel at 488 nm. The cell cycle profiles, including the G1, G2, and S, phases, and sub-G1 fractions were analyzed using CellQuest software (FACSCalibur, Becton Dickinson, Franklin Lakes, NJ, USA).

### Cellular uptake of acetylated APTS-coated Fe_3_O_4_ NPs

The cellular uptake of the acetylated APTS-coated Fe_3_O_4_ NPs was primarily evaluated by Prussian blue staining. The C6 glioma cells were plated in 12-well cell culture plates at a density of 5 × 10^5^ cells per well in RPMI 1640 medium with 10% FBS for 24 h. Following this step, the acetylated APTS-coated Fe_3_O_4_ NPs were added to each well at different concentrations (0, 10, 25, and 50 μg/mL) and incubated for 4 h at 37°C. Next, the cells were stained with Pearl's Prussian blue solution. First, the samples were treated with 4% paraformaldehyde for 10 min and were subsequently washed with Tris-NaCl buffer. The samples were subsequently exposed to Pearl's solution for 30 min before being washed with water. After that, the samples were plated onto sterile coverslips prior to microscopic imaging. The cell morphology with Prussian blue staining was observed by optical microscopy (IX71-F22FL/PH, Olympus Corp., Tokyo, Japan). The magnification was set at × 200 for all of the samples.

The cellular uptake of acetylated APTS-coated Fe_3_O_4_ NPs was further observed by TEM imaging. The C6 glioma cells were plated in six-well cell culture plates at a density of 3 × 10^5^ cells per well in RPMI 1640 medium with 10% FBS for 24 h. These cells were allowed to grow to approximately 80% confluence. Next, the acetylated APTS-coated Fe_3_O_4_ NPs were added to each well at a final concentration of 25 μg/mL and incubated for 24 h at 37°C. The culture medium was discarded, and the cells were washed with PBS buffer, trypsinized, centrifuged, washed three times with PBS buffer, and fixed with 2.5% glutaraldehyde in 0.2 M phosphate buffer (pH 7.2) for 12 h at 4°C. The cells were then post-fixed with 1% OsO_4_ in 0.2 M phosphate buffer (pH 7.2) for 2 h at 4°C. After additional washes in buffer, the cells were dehydrated and embedded with Epon 812 (Shell Chemical, UK), followed by polymerization. Next, the embedded cells were sectioned using a Reichert-Jung Ultramicrotome (Vienna, Austria). The sections with a thickness of 75 nm were mounted onto 200-mesh copper grids and counterstained with uranyl acetate and lead citrate for 5 min, respectively, prior to the TEM measurements. The grids were visualized using an H600 transmission electron microscope (Hitachi, Chiyoda-ku, Japan) with an operating voltage of 60 kV.

The intracellular uptake of APTS-coated Fe_3_O_4_ NPs in the C6 glioma cells was quantified using a Prodigy ICP-AES system (Teledyne Leeman Labs, Hudson, NH, USA). For ICP-AES analysis, 1 × 10^6^ cells were seeded onto a six-well cell culture plate for 24 h. The cells were then incubated with different concentrations of acetylated APTS-coated Fe_3_O_4_ NPs (0, 10, 25, 50, and 100 μg/mL) for 24 h. The cells were washed with PBS buffer three times, trypsinized, and harvested by centrifugation. The digestion of the cells was performed in aqua regia, and the amount of iron uptake in the cells was then quantified using ICP-AES.

### *In vitro* MR imaging of C6 glioma cells

C6 glioma cells were cultured in 10 mL RPMI 1640 that was supplemented with 10% FBS on cell culture discs, and the medium was changed every 24 to 48 h. The cells were maintained at 37°C in a humidified atmosphere with 5% CO_2_ in air. The cells were labeled with acetylated APTS-coated Fe_3_O_4_ NPs at different concentrations (10, 25, or 50 μg/mL, respectively). Next, 1 × 10^6^ labeled cells were placed into 1.5-mL Eppendorf tubes supplemented with 1 mL 1% agarose gel. An Eppendorf tube filled with 1 mL 1% agarose gel was used as a control. All of the cell phantom MR studies were performed using a Signa HDxt 3.0 T superconductor magnetic resonance system (GE Medical Systems, Milwaukee, WI, USA). An axial scan was performed using an eight-channel array head coil. *R*_2_ mapping was performed using the MFSE sequence, with a total of eight echoes and the following parameters: TR = 500 ms, TE = 21.9 ms, flip angle = 90°, resolution = 256 × 256, section thickness = 2 mm, and FOV = 80 × 80 mm. The *R*_2_ mapping reconstruction was performed by two imaging experts on a workstation running Functool 4.5.3 (GE Medical Systems, Milwaukee, WI, USA). The *R*_2_ values were calculated and recorded as the mean ± standard deviation (*n* = 3).

### *In vivo* MR imaging

Animal experiments were designed in compliance with the National Institutes of Health Guide for the Care and Use of Laboratory Animals, and the animal protocol was approved by the Institutional Animal Care and Use Committee of Shanghai First People's Hospital (Approval ID 2012-115). Sprague Dawley (SD) rats (Shanghai Slac Laboratory Animal Center, Shanghai, China) underwent surgical implantation of C6 glioma cells that were labeled with acetylated APTS-coated Fe_3_O_4_ NPs as per the following protocol. Briefly, the C6 glioma cells were cultured in RPMI 1640 that was supplemented with 10% FBS on cell culture discs and which were maintained at 37°C in a humidified atmosphere with 5% CO_2_ in air. The medium was changed every 24 to 48 h. Prior to implantation surgery, the acetylated APTS-coated Fe_3_O_4_ NPs were added into the cell culture dish at a final concentration of 25 μg/mL for an incubation of approximately 4 h. Next, the culture medium was discarded, and the cells were trypsinized, centrifuged, and washed three times with PBS buffer. In the control group, the C6 glioma cells were incubated with PBS buffer for 4 h. The SD rats were anesthetized with 1% isobarbital (3 mL/kg of body weight) delivered intraperitoneally. A sphenotresia was then performed using a stereotaxic apparatus (Gene&I, Beijing, China). One million C6 glioma cells (10 μL) that were labeled with the acetylated APTS-coated Fe_3_O_4_ NPs were injected within 10 min into the left frontal lobes of one rat (*n* = 12). In the control group, 1 × 10^6^ unlabeled C6 glioma cells were injected at a rate of 1 μL/min for 10 min into the left frontal lobe of six rats. The MR imaging (MRI) scans of the rats were performed at 7, 14, 21, and 28 days post-injection using a Signa HDxt 3.0 T superconductor magnetic resonance system (GE Medical Systems, Milwaukee, WI, USA). An axial scan was performed using a custom-built rodent receiver coil (Chenguang Med Tech, Shanghai, China). The following parameters were used for the T2-weighted images: SE/2D sequence, TR = 2,500 ms, TE = 81.9 ms, resolution = 256 × 128, section thickness = 2.4 mm, and FOV = 80 × 80 mm. The *R*_2_ mapping was performed using a MFSE sequence, with a total of eight echoes and the following parameters: TR = 500 ms, TE = 21.9 ms, flip angle = 90°, resolution = 256 × 256, section thickness = 2 mm, and FOV = 80 × 80 mm. The *R*_2_ mapping reconstruction was performed by two imaging experts on a workstation running Functool 4.5.3 (GE Medical Systems, Milwaukee, WI, USA). The *R*_2_ values in the tumor area were calculated and recorded as the mean ± standard deviation (*n* = 3) and analyzed using a two-tailed, time-dependent, paired *t* test.

### Histology study

Following the final MRI scan, each rat was deeply anesthetized, and the tumor was resected. The tumor tissues were post-fixed with 4% paraformaldehyde in a 0.1 M phosphate buffer (PB; pH 7.4) for 24 h. The samples were then dehydrated, embedded, and sectioned into 4-μm-thick slices. After dewaxing and rehydration, the tumor sections were stained with Pearl's Prussian blue solution and hematoxylin and eosin, following the manufacturer's instructions.

## Results and discussion

### The synthesis and characterization of acetylated APTS-coated Fe_3_O_4_ NPs

**Figure 1:**
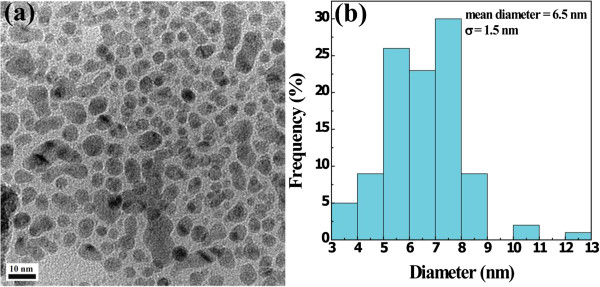
**TEM micrograph (a) and size distribution histogram (b) of acetylated APTS-coated Fe**_**3**_**O**_**4 **_**NPs.**

We obtained acetylated APTS-coated Fe_3_O_4_ NPs using the same experimental protocol as was described in our previous report [33]. TEM was utilized to characterize the synthesized acetylated APTS-coated Fe_3_O_4_ NPs (Figure 1). The TEM micrograph indicates that the particles have a spherical or quasi-spherical shape with a mean diameter of 6.5 ± 1.5 nm, in agreement with our previous results [33]. The acetylated APTS-coated Fe_3_O_4_ NPs in a powder form can be dissolved in water, PBS, or cell culture medium with good colloidal stability following storage in 4°C for a minimum of 1 month. Generally, the acetylated APTS-coated Fe_3_O_4_ NPs were stored at −20°C in a dried form before use.

### Transverse relaxivity of acetylated APTS-coated Fe_3_O_4_ NPs

**Figure 2:**
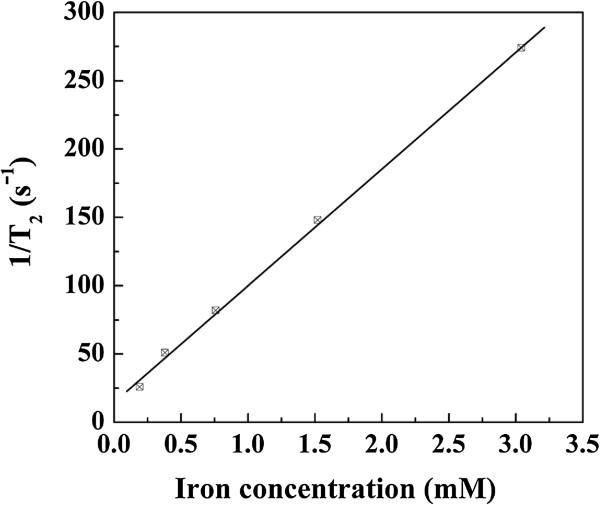
**Transverse relaxation rate (*R***_**2**_**, 1/*T***_**2**_**) for acetylated APTS-coated Fe**_**3**_**O**_**4 **_**NPs as a function of Fe concentration.**

The magnetic behavior of Fe_3_O_4_-based NPs is very important for their biomedical applications. The transverse relaxation time (*T*_2_) of the NPs was measured to evaluate the possibility of using acetylated APTS-coated Fe_3_O_4_ NPs as a potential *T*_2_-based contrast agent for MR imaging. The measured *T*_2_ data were used to calculate the transverse relaxivity (*R*_2_) (the transverse relaxation rate per millimolar of iron), which represents the efficiency of NPs as a *T*_2_ contrast agent. As is shown in Figure 2, the transverse relaxation rate (*R*_2_ = 81.5 mM^−1^ s^−1^) as a function of the Fe concentration indicates that the relaxation rate increases linearly with the Fe concentration with a slope that is larger than that of Fe_3_O_4_ NPs coated with polymer multilayers (*R*_2_ = 78.8 mM^−1^ s^−1^) [31]. Our results suggest that acetylated APTS-coated Fe_3_O_4_ NPs may be used as a *T*_2_-shortening agent, due to their small size and relatively large *R*_2_ value.

### The cytotoxicity of acetylated APTS-coated Fe_3_O_4_ NPs

**Figure 3:**
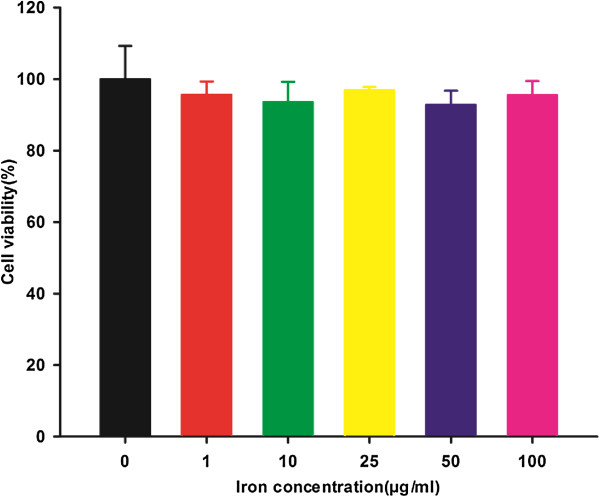
**MTT assay of C6 glioma cell viability following treatment with acetylated APTS-coated Fe**_**3**_**O**_**4 **_**NPs for 24 h.** The mean and the SEM for the triplicate wells are reported. The data are expressed as the mean ± SEM.

The MTT assay was used to assess the viability of C6 glioma cells that were treated with acetylated APTS-coated Fe_3_O_4_ NPs (Figure 3). Compared to the PBS control, there was no statistically significant difference in the viability of cells that were treated with the particles at a concentration range of 0 to 100 μg/mL (*p* > 0.05), suggesting that the acetylated APTS-coated Fe_3_O_4_ NPs are noncytotoxic at the given concentration range.

**Figure 4:**
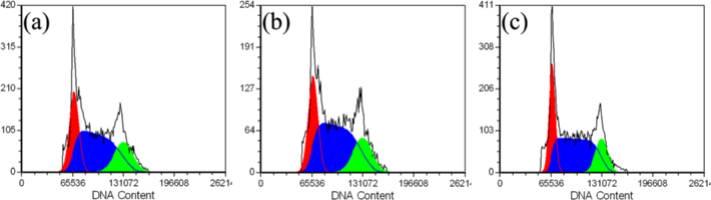
**Flow cytometry analysis.** Flow cytometry analysis of C6 glioma cells that were treated with the acetylated APTS-coated Fe_3_O_4_ NPs at concentrations of **(a)** 50 μg/mL and **(b)** 100 μg/mL for 4 h at 37°C (*n* = 4). The data of the untreated negative control cells is shown in **(c)**. Red, G1 phase; blue, S phase; green, G2 phase.

Cell cycle damage is one of the most important features of cytotoxicity [35]. The cell phase distribution is generally analyzed by the determination of DNA content, and the fraction of DNA content in the sub-G1 phase is an indicator of apoptosis [36,37]. To investigate further the influence of the acetylated APTS-coated Fe_3_O_4_ NPs on apoptosis, the treated cells were analyzed using flow cytometry. The sub-G1 fraction of C6 glioma cells that were incubated with acetylated APTS-coated Fe_3_O_4_ NPs at concentrations of 50 and 100 μg/mL were determined to be 2.38% ± 0.29% and 2.40% ± 0.33% (Table 1), respectively, with no statistically significant difference compared to the PBS-treated control cells (2.39% ± 0.14%, *p* > 0.05). This result also demonstrates that acetylated APTS-coated Fe_3_O_4_ NPs have no effect on the cell cycle of C6 glioma cells (Figure 4, Table 1).

**Table 1:** **Apoptosis and cell cycle analysis of C6 glioma cells following incubation with Fe**_**3**_**O**_**4 **_**NPs for 4 h**

**Group**	**Apoptosis (%)**	**Cell cycle (%)**			
		**G1**	**G2**	**S**	**G2/G1**
Control	2.39 ± 0.14	27.32 ± 0.45	19.42 ± 0.07	53.27 ± 0.33	1.93 ± 0.01
50 μg/mL	2.38 ± 0.29	27.22 ± 0.43	18.74 ± 0.12	54.05 ± 0.39	1.93 ± 0.02
100 μg/mL	2.40 ± 0.33	27.38 ± 0.52	18.64 ± 0.13	55.02 ± 0.41	1.93 ± 0.01

Mean ± standard deviation, *n* = 4.

### The *in vitro* cellular uptake of acetylated APTS-coated Fe_3_O_4_ NPs

**Figure 5:**
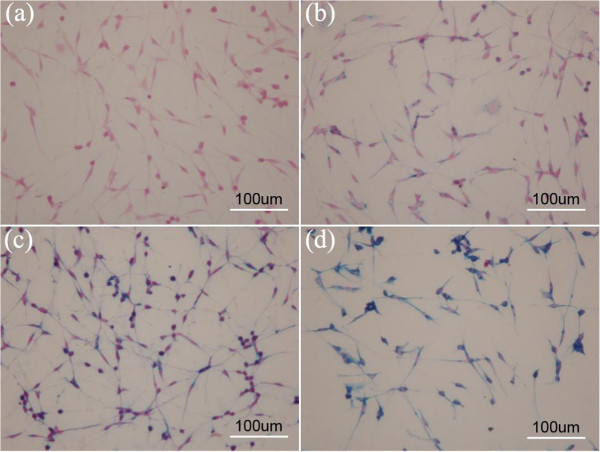
**Optical microscopic images of C6 glioma cells.** Prussian blue staining of C6 glioma cells that were treated with PBS buffer **(a)** and those that were treated with acetylated APTS-coated Fe_3_O_4_ NPs at a concentration of 10 μg/mL **(b)**, 25 μg/mL **(c)**, and 50 μg/mL **(d)** (scale bar = 100 μm).

To determine the cellular uptake of the APTS-coated Fe_3_O_4_ NPs, the C6 glioma cells that were incubated with the particles for 24 h were stained with Prussian blue and imaged with optical microscopy (Figure 5). The C6 glioma cells that were labeled with higher concentrations (25 and 50 μg/mL) clearly exhibited deeper blue staining than either those that were labeled using a less concentrated particle solution (10 μg/mL) or untreated control cells, indicating the higher intracellular uptake of the Fe_3_O_4_ NPs. Moreover, the Prussian blue staining data also indicate that the incubation of the acetylated APTS-coated Fe_3_O_4_ NPs at a concentration as high as 50 μg/mL does not markedly affect the regular spindle-shaped cell morphology when compared to the PBS control; this result is in agreement with the MTT cell viability assay data.

**Figure 6:**
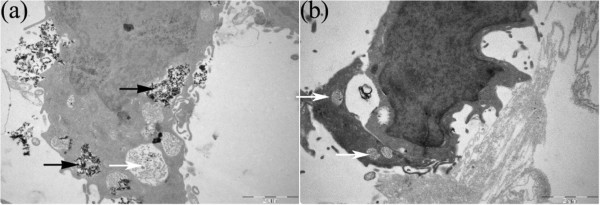
**TEM images.** TEM images of C6 glioma cells that were incubated with the acetylated APTS-coated Fe_3_O_4_ NPs at a concentration of 25 μg/mL for 24 h **(a)** and C6 glioma cells that were treated with PBS buffer **(b)**. The acetylated APTS-coated Fe_3_O_4_ NPs in the endosomes are visible as electron-dense nanoparticles and are indicated by black arrows. The white arrows indicate the normal endosome without NPs.

The C6 glioma cells that were treated with the acetylated APTS-coated Fe_3_O_4_ NPs were also imaged by TEM to identify the uptake of the particles (Figure 6). Numerous electron-dense particles can be observed in the cytoplasm of the C6 glioma cells following incubation with acetylated APTS-coated Fe_3_O_4_ NPs for 24 h. In contrast, control cells that were not treated with the NPs do not exhibit such high electron-dense particles. The TEM studies suggest that acetylated APTS-coated Fe_3_O_4_ NPs are able to be taken up by the C6 glioma cells.

**Figure 7:**
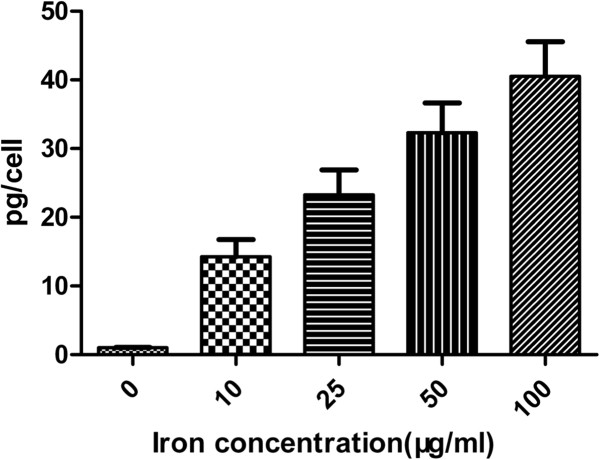
**Intracellular uptake.** The intracellular uptake of acetylated APTS-coated Fe_3_O_4_ NPs quantified using ICP-AES after the C6 glioma cells were treated with the particles at different concentrations for 24 h.

The cellular uptake of acetylated APTS-coated Fe_3_O_4_ NPs was further quantified using ICP-AES (Figure 7). It is clear that iron uptake in C6 glioma cells increases approximately linearly with the particle concentration. The ICP-AES data corroborate the Prussian blue staining results. Overall, the Prussian blue staining, TEM imaging, and ICP-AES results together confirmed the intracellular uptake of the acetylated APTS-coated Fe_3_O_4_ NPs, which is essential for them to be used for cancer cell imaging.

### The *in vitro* MR detection of C6 glioma cells

**Figure 8:**
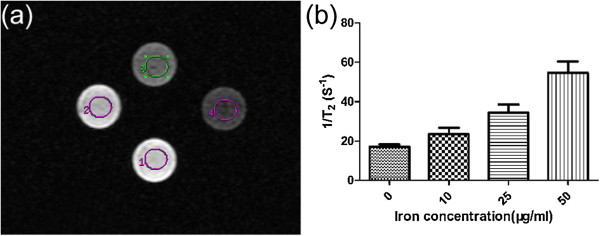
***R***_**2**_**mapping and*R***_**2**_**values of the C6 glioma cell phantoms. (a)***R*_2_ mapping of gel phantoms containing C6 glioma cells that were treated with PBS buffer (1) or with acetylated APTS-coated Fe_3_O_4_ NPs at concentrations of 10 μg/mL (2), 25 μg/mL (3), and 50 μg/mL (4). **(b)***R*_2_ values of the C6 glioma cells with the above treatments.

To conclusively demonstrate our hypothesis that acetylated APTS-coated Fe_3_O_4_ NPs can be used as an effective molecular imaging labeling agent via MR imaging, C6 glioma cells that were treated with different concentrations of NPs (0, 10, 25, and 50 μg/mL, respectively) were imaged using a 3.0-T MR imaging system (Figure 8). The transverse MR images of C6 glioma cells that were incubated with the acetylated APTS-coated Fe_3_O_4_ NPs reveal that the cells gradually become darker with increasing particle concentrations (Figure 8a). A further quantitative analysis of the transverse relaxivities (*R*_2_, 1/*T*_2_) of the cells (Figure 8b) indicated that the *R*_2_ of C6 glioma cells that were incubated with the acetylated APTS-coated Fe_3_O_4_ NPs at a concentration of 100 μg/mL was significantly higher than those of the cells that were incubated with lower concentrations of particles (10 and 25 μg/mL) and that of the negative control cells (*p* < 0.05). These results suggest that acetylated APTS-coated Fe_3_O_4_ NPs that are taken up by the cells are able to hamper the MR signal intensity of the cells, thereby enabling effective MR detection of cancer cells *in vitro*.

### *In vivo* MR imaging of xenografted C6 glioma tumor model

**Figure 9:**
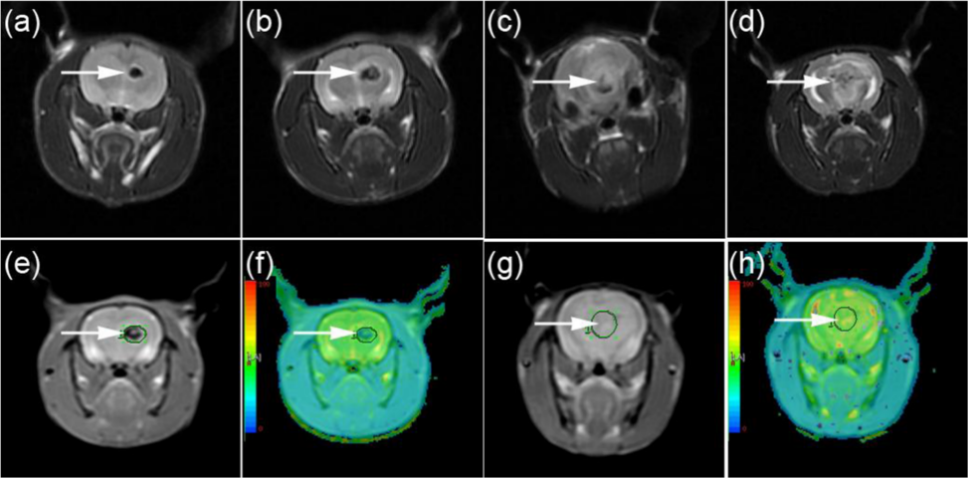
**MR imaging of C6 glioma xenograft tumor model.** T2-weighted MR images of C6 glioma xenografts that were labeled with 25 μg/mL acetylated APTS-coated Fe_3_O_4_ NPs at **(a)** 7 days, **(b)** 14 days, **(c)** 21 days, and **(d)** 28 days. **(e)** The *R*_2_ mapping of C6 glioma xenografts that were labeled with 25 μg/mL acetylated APTS-coated Fe_3_O_4_ NPs at 14 days. **(f)** A pseudocolor picture of (e). **(g)** The *R*_2_ mapping of C6 glioma xenografts without labeling at 14 days as a control. **(h)** A pseudocolor photo of **(g)**. The white arrows indicate the glioma xenografts.

**Figure 10:**
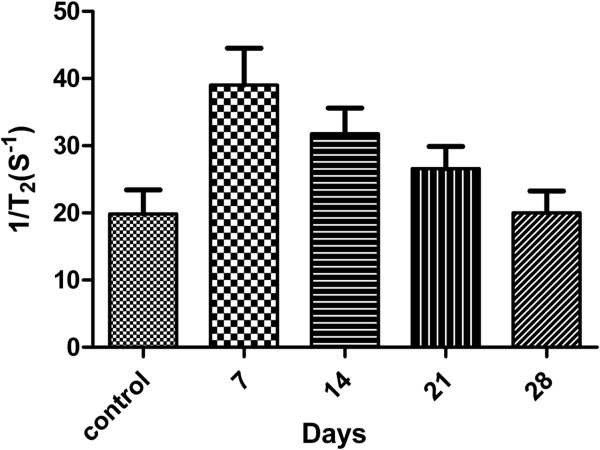
***R***_**2**_**values of C6 glioma xenografts labeled with 25 μg/mL Fe**_**3**_**O**_**4**_**NPs at 7, 14, 21, and 28 days.** The *R*_2_ value of C6 glioma xenografts that were treated with PBS buffer after 14 days was used as a control value.

The excellent *in vitro* performance of the acetylated APTS-coated Fe_3_O_4_ NPs for C6 glioma cell MR imaging in addition to the excellent biocompatibility of the particles encouraged us to pursue the applicability of these NPs for the *in vivo* MR imaging study in SD rats. Figure 9 clearly illustrates that the C6 glioma cells that were labeled with the acetylated APTS-coated Fe_3_O_4_ NPs exhibited a clear contrast in the tumor area, with a significantly lower signal intensity when compared to unlabeled C6 glioma cells. Moreover, following analyses at different time points, we determined that the *R*_2_ value of the tumor area labeled with the acetylated APTS-coated Fe_3_O_4_ NPs decreased gradually with time. However, at 21 days following the intracranial injection of the NP-labeled C6 glioma cells, the *R*_2_ value of the tumor area was significantly higher than that of the unlabeled tumor area (Figure 10). These results clearly indicate that acetylated APTS-coated Fe_3_O_4_ NPs enable the MR detection of C6 glioma cells both *in vitro* and *in vivo*.

**Figure 11:**
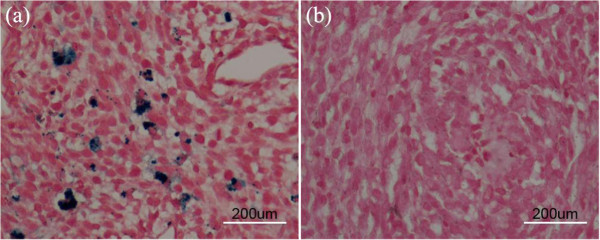
**Prussian blue staining of C6 glioma xenografts on the 14th day. (a)** The tumor model was labeled with 25 μg/mL of acetylated APTS-coated Fe_3_O_4_ NPs (scale bar = 200 μm). **(b)** A negative PBS control without particle labeling (scale bar = 200 μm).

To confirm further the localization of the acetylated APTS-coated Fe_3_O_4_ NPs in the tumor site, the tumor sections were stained using Prussian blue and observed using an optical microscope (Figure 11). In the sections of the NP-labeled xenografted tumors that were isolated 14 days following the injection of the C6 glioma cells, numerous blue spots were observed to clearly localize in the cytoplasm of the cells, indicating the presence of the Fe_3_O_4_ NPs (Figure 11a). In contrast, no blue spots were observed in the negative control (Figure 11b). Our results suggest that the acetylated APTS-coated Fe_3_O_4_ NPs can be retained in the tumor site for a comparatively long time, allowing effective MR imaging of tumors.

## Conclusions

In summary, we developed a novel type of acetylated APTS-coated Fe_3_O_4_ NPs with a mean diameter of 6.5 nm for MR imaging both *in vitro* and *in vivo*. Combined morphological observation of cells, MTT assays of cell viability, and flow cytometric analyses of cell cycle characteristics indicate that acetylated APTS-coated Fe_3_O_4_ NPs do not appreciably affect the cell morphology, viability, or the cell cycle, indicating their good biocompatibility at the given concentration range. Furthermore, Prussian blue staining of cell morphology, TEM imaging, and ICP-AES quantification data indicate that acetylated APTS-coated Fe_3_O_4_ NPs are able to be taken up by cells in a concentration-dependent manner. The intracellular uptake of the particles enables effective MR imaging of model tumor cells (e.g., C6 glioma cells) *in vitro* and in the xenograft tumor model *in vivo*. Moreover, given the relatively high transverse relaxivity and the tunable amine chemistry of APTS-coated Fe_3_O_4_ NPs, which can be further functionalized with various targeting ligands (e.g., folic acid and RGD peptides), it is expected that such NPs may be further biofunctionalized for various biomedical applications, especially for targeted MR imaging.

## Competing interests

The authors declare that they have no competing interests.

## Authors’ contributions

KL, MS, XS, and GZ carried out the conception and design of this study. MS and XS carried out the design of the nanoparticles studies and participated in the synthesis and characterization of the acetylated APTS-coated Fe_3_O_4_ NPs. KL, LZ, QQ, JZ, and GZ carried out the *in vitro* and *in vivo* studies and participated in the cell culture, cytotoxity, and imaging studies. KL performed the statistical analysis. All authors carried out the manuscript drafting. All authors read and approved the final manuscript.

## References

[B1] Arbab AS Janic B Haller J Pawelczyk E Liu W Frank JA In vivo cellular imaging for translational medical research Curr Med Imaging Rev 2009 9 19 38 10.2174/157340509787354697 19768136 PMC2746660

[B2] Artemov D Mori N Ravi R Bhujwalla ZM Magnetic resonance molecular imaging of the HER-2/neu receptor Cancer Res 2003 9 2723 2727 12782573

[B3] Moore A Medarova Z Potthast A Dai G In vivo targeting of underglycosylated MUC-1 tumor antigen using a multimodal imaging probe Cancer Res 2004 9 1821 1827 10.1158/0008-5472.CAN-03-3230 14996745

[B4] Wang J Xie J Zhou X Cheng Z Gu N Teng G Hu Q Zhu F Chang S Zhang F Lu G Chen X Ferritin enhances SPIO tracking of C6 rat glioma cells by MRI Mol Imaging Biol 2011 9 87 93 10.1007/s11307-010-0338-5 20440566 PMC2966504

[B5] Arbab AS Yocum GT Wilson LB Parwana A Jordan EK Kalish H Frank JA Comparison of transfection agents in forming complexes with ferumoxides, cell labeling efficiency, and cellular viability Mol Imaging 2004 9 24 32 10.1162/153535004773861697 15142409

[B6] Zhang Z Dharmakumar R Mascheri N Fan Z Wu S Li D Comparison of superparamagnetic and ultrasmall superparamagnetic iron oxide cell labeling for tracking green fluorescent protein gene marker with negative and positive contrast magnetic resonance imaging Mol Imaging 2009 9 148 155 19723472 PMC2847689

[B7] Balakumaran A Pawelczyk E Ren J Sworder B Chaudhry A Sabatino M Stroncek D Frank JA Robey PG Superparamagnetic iron oxide nanoparticles labeling of bone marrow stromal (mesenchymal) cells does not affect their “stemness” PLoS One 2010 9 e11462 10.1371/journal.pone.0011462 20628641 PMC2898800

[B8] Arnold LJ Jr Dagan A Gutheil J Kaplan NO Antineoplastic activity of poly(L-lysine) with some ascites tumor cells Proc Natl Acad Sci U S A 1979 9 3246 3250 10.1073/pnas.76.7.3246 291000 PMC383801

[B9] Xie J Chen K Lee H-Y Xu C Hsu AR Peng S Chen X Sun S Ultrasmall c(RGDyK)-coated Fe3O4 nanoparticles and their specific targeting to integrin alpha(v)beta3-rich tumor cells J Am Chem Soc 2008 9 7542 7543 10.1021/ja802003h 18500805 PMC2542944

[B10] Kraitchman DL Gilson WD Lorenz CH Stem cell therapy: MRI guidance and monitoring J Magn Reson Imaging 2008 9 299 310 10.1002/jmri.21263 18219684 PMC3075622

[B11] Cohen ME Muja N Fainstein N Bulte JW Ben-Hur T Conserved fate and function of ferumoxides-labeled neural precursor cells in vitro and in vivo J Neurosci Res 2010 9 936 944 19885865 10.1002/jnr.22277 PMC3031987

[B12] Kim H Walczak P Muja N Campanelli JT Bulte JW ICV-transplanted human glial precursor cells are short-lived yet exert immunomodulatory effects in mice with EAE Glia 2012 9 1117 1129 10.1002/glia.22339 22499166 PMC3579214

[B13] Neri M Maderna C Cavazzin C Deidda-Vigoriti V Politi LS Scotti G Marzola P Sbarbati A Vescovi AL Gritti A Efficient in vitro labeling of human neural precursor cells with superparamagnetic iron oxide particles: relevance for in vivo cell tracking Stem Cells 2008 9 505 516 10.1634/stemcells.2007-0251 17975226

[B14] Pawelczyk E Arbab AS Pandit S Hu E Frank JA Expression of transferrin receptor and ferritin following ferumoxides-protamine sulfate labeling of cells: implications for cellular magnetic resonance imaging NMR Biomed 2006 9 581 592 10.1002/nbm.1038 16673357

[B15] Wang SH Shi XY Van Antwerp M Cao ZY Swanson SD Bi XD Baker JR Jr Dendrimer-functionalized iron oxide nanoparticles for specific targeting and imaging of cancer cells Adv Funct Mater 2007 9 3043 3050 10.1002/adfm.200601139

[B16] Gupta AK Gupta M Synthesis and surface engineering of iron oxide nanoparticles for biomedical applications Biomaterials 2005 9 3995 4021 10.1016/j.biomaterials.2004.10.012 15626447

[B17] Gass J Poddar P Almand J Srinath S Srikanth H Superparamagnetic polymer nanocomposites with uniform Fe_3_O_4_ nanoparticle dispersions Adv Funct Mater 2006 9 71 75 10.1002/adfm.200500335

[B18] Iida H Nakanishi T Takada H Osaka T Preparation of magnetic iron-oxide nanoparticles by successive reduction-oxidation in reverse micelles: effects of reducing agent and atmosphere Electrochim Acta 2006 9 292 296 10.1016/j.electacta.2006.05.007

[B19] Sun SH Zeng H Size-controlled synthesis of magnetite nanoparticles J Am Chem Soc 2002 9 8204 8205 10.1021/ja026501x 12105897

[B20] Ge S Shi XY Sun K Li CP Uher C Baker JR Jr Holl MMB Orr BG Facile hydrothermal synthesis of iron oxide nanoparticles with tunable magnetic properties J Phys Chem C 2009 9 13593 13599 10.1021/jp902953t PMC2823489 20174618

[B21] Feng J Mao J Wen XG Tu MJ Ultrasonic-assisted in situ synthesis and characterization of superparamagnetic Fe_3_O_4_ nanoparticles J Alloy Compd 2011 9 9093 9097 10.1016/j.jallcom.2011.06.053

[B22] Xu YL Qin Y Palchoudhury S Bao YP Water-soluble iron oxide nanoparticles with high stability and selective surface functionality Langmuir 2011 9 8990 8997 10.1021/la201652h 21644795

[B23] Giri S Trewyn BG Stellmaker MP Lin VSY Stimuli-responsive controlled-release delivery system based on mesoporous silica nanorods capped with magnetic nanoparticles Angew Chem Int Ed 2005 9 5038 5044 10.1002/anie.200501819 16038000

[B24] Mohapatra S Pramanik N Mukherjee S Ghosh SK Pramanik P A simple synthesis of amine-derivatised superparamagnetic iron oxide nanoparticles for bioapplications J Mater Sci 2007 9 7566 7574 10.1007/s10853-007-1597-7

[B25] Wu W He Q Jiang C Magnetic iron oxide nanoparticles: synthesis and surface functionalization strategies Nanoscale Res Lett 2008 9 397 415 10.1007/s11671-008-9174-9 21749733 PMC3244954

[B26] Cheng L Yang K Li Y Chen J Wang C Shao M Lee S-T Liu Z Facile preparation of multifunctional upconversion nanoprobes for multimodal imaging and dual-targeted photothermal therapy Angew Chem Int Ed 2011 9 7385 7390 10.1002/anie.201101447 21714049

[B27] Huh Y-M Jun Y-w Song H-T Kim S Choi J-s Lee J-H Yoon S Kim K-S Shin J-S Suh J-S Cheon J In vivo magnetic resonance detection of cancer by using multifunctional magnetic nanocrystals J Am Chem Soc 2005 9 12387 12391 10.1021/ja052337c 16131220

[B28] Song H-T Choi J-s Huh Y-M Kim S Jun Y-w Suh J-S Cheon J Surface modulation of magnetic nanocrystals in the development of highly efficient magnetic resonance probes for intracellular labeling J Am Chem Soc 2005 9 9992 9993 10.1021/ja051833y 16011350

[B29] Hu FQ Li Z Tu CF Gao MY Preparation of magnetite nanocrystals with surface reactive moieties by one-pot reaction J Colloid Interf Sci 2007 9 469 474 10.1016/j.jcis.2007.03.023 17433352

[B30] Hu FQ Wei L Zhou Z Ran YL Li Z Gao MY Preparation of biocompatible magnetite nanocrystals for in vivo magnetic resonance detection of cancer Adv Mater 2006 9 2553 2556 10.1002/adma.200600385

[B31] Shen M Shi X Dendrimer-based organic/inorganic hybrid nanoparticles in biomedical applications Nanoscale 2010 9 1596 1610 10.1039/c0nr00072h 20820690

[B32] Shi XY Wang SH Swanson SD Ge S Cao ZY Van Antwerp ME Landmark KJ Baker JR Jr Dendrimer-functionalized shell-crosslinked iron oxide nanoparticles for in-vivo magnetic resonance imaging of tumors Adv Mater 2008 9 1671 1678 10.1002/adma.200702770

[B33] Shen M Cai H Wang X Cao X Li K Wang SH Guo R Zheng L Zhang G Shi X Facile one-pot preparation, surface functionalization, and toxicity assay of APTS-coated iron oxide nanoparticles Nanotechnology 2012 9 105601 10.1088/0957-4484/23/10/105601 22349004

[B34] Peng C Li K Cao X Xiao T Hou W Zheng L Guo R Shen M Zhang G Shi X Facile formation of dendrimer-stabilized gold nanoparticles modified with diatrizoic acid for enhanced computed tomography imaging applications Nanoscale 2012 9 6768 6778 10.1039/c2nr31687k 23010987

[B35] Smith JA Martin L Do cells cycle? Proc Natl Acad Sci U S A 1973 9 1263 1267 10.1073/pnas.70.4.1263 4515625 PMC433472

[B36] Dolbeare F Gratzner H Pallavicini MG Gray JW Flow cytometric measurement of total DNA content and incorporated bromodeoxyuridine Proc Natl Acad Sci U S A 1983 9 5573 5577 10.1073/pnas.80.18.5573 6577444 PMC384300

[B37] Kajstura M Halicka HD Pryjma J Darzynkiewicz Z Discontinuous fragmentation of nuclear DNA during apoptosis revealed by discrete “sub-G1” peaks on DNA content histograms Cytometry A 2007 9 125 131 17252584 10.1002/cyto.a.20357

